# Metabolic-immune crosstalk in head and neck squamous cell carcinoma: CD44 and APP identified as causal therapeutic targets via integrated lactylation-Mendelian randomization analysis

**DOI:** 10.3389/fimmu.2026.1808455

**Published:** 2026-05-28

**Authors:** Xi Zhang, Qicheng Deng, Ling Yang, Min Yan

**Affiliations:** 1 Department of Otolaryngology Head and Neck Surgery, Beijing Anzhen Hospital of Capital Medical University & Nanchong Central Hospital, Nanchong, Sichuan, China; 2 Public Health Clinical Center of Chengdu, Chengdu, Sichuan, China; 3 Department of Otolaryngology Head and Neck Surgery, Dazhou Central Hospital, Dazhou, Sichuan, China; 4 Beijing Anzhen Hospital of Capital Medical University & Nanchong Central Hospital, Nanchong, Sichuan, China

**Keywords:** APP, CD44, head and neck squamous cell carcinoma, lactylation, Mendelian randomization, metabolic-immune crosstalk

## Abstract

**Background:**

Patients with head and neck squamous cell carcinoma (HNSCC) continue to face poor prognosis, highlighting an urgent need for new diagnostic markers and therapeutic targets. While metabolic reprogramming and immune microenvironment dysregulation are crucial drivers of HNSCC progression, the key causal molecular mechanisms linking these processes remain elusive. Post-translational modifications, especially protein lactylation, may serve as a vital interface for this metabolic-immune “crosstalk”.

**Methods:**

We developed an integrative analytical framework merging lactylation proteomics, transcriptomics, and Mendelian randomization (MR). Differential expression analysis was conducted on three public transcriptomic cohorts (53 HNSCC vs. 53 controls), and the resulting genes were overlapped with a systematically compiled set of 2, 124 lactylation-related genes. Causal risk genes were then identified using MR analysis with large-scale genetic instruments (from expression quantitative trait locus data) and HNSCC genome-wide association study summary statistics. The functional roles of candidate genes were explored through enrichment analysis, Gene Set Variation Analysis, and immune deconvolution (CIBERSORT). Experimental validation was performed using quantitative real-time PCR and Western blotting in an independent The Cancer Genome Atlas dataset and in HNSCC cell lines.

**Results:**

We identified 212 lactylation-associated differentially expressed genes. MR analysis established CD44 and APP as genetic causal risk factors for HNSCC, with both genes significantly overexpressed in patient tissues. Functional profiling indicated that high CD44 expression correlated with activation of mTOR signaling and ECM-receptor interaction pathways, and was positively associated with M0 macrophage infiltration. Conversely, high APP expression was linked to activated protein secretion and ECM pathways, and showed a positive correlation with M2 macrophage abundance. The marked upregulation of CD44 and APP in HNSCC was consistently confirmed in the independent validation cohort and in cellular models.

**Conclusion:**

By pioneering a multi-omics causal inference approach in HNSCC, this study identifies CD44 and APP as genetic causal risk factors for disease susceptibility and progression. These genes connect distinct metabolic pathways with specific immune cell subsets, functioning as central hubs within the HNSCC metabolic-immune crosstalk network. Our work provides a critical theoretical basis for future development of lactylation pathway-based biomarkers and targeted interventions.

## 1 Introduction

Head and neck cancer is the seventh most prevalent malignancy globally. The 2022 global cancer statistics reported an estimated 890, 000 new cases and 460, 000 deaths, representing about 4.5% of all new cancer diagnoses and 4.7% of all cancer-related mortality, respectively (1, 2). The most common pathological type is head and neck squamous cell carcinoma (HNSCC), which originates predominantly from the mucosal epithelium of the oral cavity, larynx, and pharynx, and comprises roughly 90% of head and neck cancer cases (3). Key risk factors for HNSCC include tobacco use, alcohol consumption, and human papillomavirus (HPV) infection (4, 5). Although multimodal treatment strategies for HNSCC have evolved considerably in recent years, encompassing surgery, radiation, targeted agents, and immunotherapy, recurrence or metastasis occurs in more than half of patients, and the worldwide 5-year overall survival rate persists under 60% (6, 7). In advanced-stage disease, the 5-year overall survival rate is merely around 50% (3). Chemotherapy and immunotherapy resistance also significantly contribute to the poor survival outcomes in HNSCC (8–10). Consequently, uncovering the molecular mechanisms driving HNSCC development and progression is of paramount importance for enhancing patient prognosis.

Tumor cells preferentially engage in glycolysis even in the presence of oxygen, a metabolic adaptation termed the Warburg effect, resulting in substantial lactate production (11–13). Once considered merely a metabolic byproduct, lactate is now recognized as a signaling molecule driving tumor metastasis, angiogenesis, and immunosuppression within the tumor microenvironment (TME) (14). Specifically in HNSCC, lactate promotes cancer stemness and type I collagen deposition (11). Beyond these classical functions, a recent breakthrough has revealed that lactate can also serve as a substrate for a novel post-translational modification—histone lysine lactylation—thereby directly linking metabolic state to epigenetic gene regulation (15–17). In HNSCC, emerging evidence demonstrates that this lactate-driven epigenetic reprogramming can induce CD8+ T cell exhaustion, suggesting a mechanistic link between high histone lactylation levels and suboptimal immunotherapy responses (18). Consequently, the identification of lactylation-associated targets in HNSCC is of substantial clinical relevance for therapeutic development.

While transcriptomic investigations in human HNSCC uniformly reveal dysregulated energy metabolism and immune features (19), the integration of transcriptomics, lactylation proteomics, and causal inference methodologies to pinpoint causal regulatory factors remains unexplored. Traditional differential expression gene (DEG) analysis is inherently correlative and cannot infer causation, and isolated studies on post-translational modifications (PTMs) often lack strong genetic support for their clinical significance. Thus, two critical questions impede further advancement (1): Which lactylation-related DEGs possess a causal role in elevating HNSCC risk? (2) Through what mechanisms do these genes dualistically mediate both metabolic dysfunction and immune microenvironment reshaping?

Mendelian randomization (MR) provides a powerful method to tackle the aforementioned issues, employing genetic variants as instrumental variables (IVs) for causal inference (20). Nonetheless, the combined use of lactylation proteomics, multi-cohort transcriptomics, and MR remains unexplored in HNSCC research. This methodological void significantly limits the advancement of treatment strategies. In essence, promising HNSCC interventions must fulfill two key criteria: they should be underpinned by genetic causal evidence and exert dual regulatory functions at the intersection of metabolic and immune pathways—attributes that are beyond the reach of any single-omics approach. Lacking a comprehensive integrative framework, the diagnostic and therapeutic promise of lactylation-linked DEGs cannot be fully realized. To address this deficiency, we developed an integrated analytical framework that merges lactylation modification with MR. Through the analysis of three HNSCC transcriptomic cohorts (totaling 53 HNSCC samples and 53 normal controls), integrated with lactylation proteomics, DEG screening, and MR, we pinpointed HNSCC risk genes with causal links and experimentally confirmed their involvement in metabolic-immune dysregulation and the dynamic regulation of the immune microenvironment. Our work pioneers the identification of CD44 and APP as causal regulators in HNSCC pathogenesis that are associated with lactylation modification, thereby establishing a rationale for their diagnostic utility and therapeutic prospects.

## 2 Materials and methods

### 2.1 Data collection

Human gene expression profiles were acquired from the Gene Expression Omnibus (GEO) database by searching for “HNSCC” and “Homo sapiens”. Datasets were selected according to the following inclusion criteria: (1) a minimum of 10 samples per individual dataset; (2) availability of at least 5 HNSCC tumor samples alongside 5 matched normal control or adjacent normal tissue samples; (3) absence of any prior chemical or genetic manipulation of the samples; (4) provision of either raw data or a normalized expression matrix. Following these criteria, three datasets (GSE6631, GSE13399, GSE58911) were selected, encompassing a total of 53 HNSCC and 53 control samples. For independent validation, data were downloaded from The Cancer Genome Atlas (TCGA) database (https://portal.gdc.cancer.gov/), consisting of 515 HNSCC tumor tissues and 44 non-tumor tissues. The detailed features of all datasets are presented in **Table 1**.

**Table 1:** The information of the dataset source in this study.

ID	Data type	Contains
GSE6631	RNA-seq database	22 tumors and 22 normal
GSE13399	RNA-seq database	16 tumors and 16 normal
GSE58911	RNA-seq database	15 tumors and 15 normal
TCGA-HNSC	RNA-seq database	515 tumors and 44 normal
ieu-b-4912	GWAS	1, 106 tumors and 372, 016 normal

### 2.2 DEG identification

All statistical analyses were carried out using R (version 4.5.2). Background correction and quantile normalization of the raw expression data were implemented with the limma package. Principal Component Analysis (PCA) was employed to evaluate and adjust for inter-dataset batch effects. For identifying DEGs, a linear model fitted with empirical Bayes moderation was applied, with significance thresholds set at P < 0.05 and |log_2_fold change (FC)| > 0.585 (21). Applying these thresholds yielded 1128 DEGs (**Supplementary Table 1**).

### 2.3 Lactylation-related genes

Lactylation-related genes (LRGs) were compiled from a systematic review of published literature and public databases, notably PubMed (https://pubmed.ncbi.nlm.nih.gov/) and GeneCards (https://www.genecards.org/), with “lactylation” as the search term (22–30). This process yielded a curated list of 2, 124 LRGs (**Supplementary Table 2**). The overlap between this LRG list and the set of DEGs identified in our study was then determined, resulting in 212 lactylation-associated DEGs (**Supplementary Table 3**).

### 2.4 MR analysis

Genetic variants showing significant associations with the expression of lactylation-associated DEGs served as IVs in this study. These variants (single nucleotide polymorphisms, SNPs) were derived from a dedicated expression quantitative trait locus database, filtered at a significance threshold of P < 5 × 10–^6^ for gene expression association (31, 32). To guarantee instrument independence, the initially identified SNPs underwent linkage disequilibrium clumping analysis (with parameters r² < 0.001 and a distance window of 10, 000 kb). Subsequently, weak instruments with an F-statistic below 10 were removed (33, 34), yielding a final set of 42, 699 robust instrumental SNPs for subsequent analyses (**Supplementary Table 4**).

Summary statistics from a head and neck cancer genome-wide association study (GWAS) were utilized as the outcome data, specifically from the ieu-b-4912 dataset (1, 106 cases and 372, 016 controls, all of European descent) (35). Following harmonization of the genetic data for the exposure (gene expression) and the outcome (HNSCC risk), the inverse-variance weighted (IVW) method was applied as the primary approach for causal estimation. To assess the robustness of the results, heterogeneity was evaluated using the MR-Egger method, and sensitivity analysis was performed using the leave-one-out approach. Additionally, the presence of horizontal pleiotropy was tested via the intercept of the MR-Egger regression model, with a non-significant intercept (P > 0.05) indicating that significant pleiotropic bias was unlikely.

### 2.5 Protein-protein interaction network analysis

To identify hub genes and functional modules among the 212 lactylation-associated DEGs, a protein–protein interaction (PPI) network was constructed using the STRING database (version 11.5) with a confidence score ≥ 0.7. The network was visualized and analyzed using Cytoscape (version 3.9.1). Hub genes were defined as nodes with high connectivity, and CD44 and APP were evaluated for their centrality within the network.

### 2.6 Functional enrichment

To explore the biological roles of the lactylation-associated DEGs, we conducted functional enrichment analysis on the set of 212 genes using the clusterProfiler package in R. The analysis covered both Gene Ontology (GO) annotations—spanning Biological Processes, Molecular Functions, and Cellular Components—and pathways from the Kyoto Encyclopedia of Genes and Genomes (KEGG). Enriched terms and pathways were considered significant at a False Discovery Rate-adjusted P-value of less than 0.05.

Additionally, Gene Set Variation Analysis (GSVA) was conducted to examine how the expression levels of the hub genes (CD44 and APP) influence biological pathway activities. Samples were stratified into a high-expression cohort (expression ≥ median) and a low-expression cohort (expression < median) based on their expression values, and pathway activity scores were then compared between these groups. This stratification approach, akin to the methodology used in comparable studies, was employed to robustly detect pathway changes linked to differential gene expression.

### 2.7 Immune cell infiltration

To characterize the immune cell landscape in the HNSCC TME, we utilized the CIBERSORT algorithm with its default LM22 signature matrix to deconvolute the transcriptomic profiles, estimating the relative abundances of 22 distinct immune cell subtypes. For cross-validation, immune infiltration was also analyzed using the xCell algorithm, which enables sensitive and robust quantification of 64 immune and stromal cell types. Furthermore, Spearman’s rank correlation analysis was applied to assess associations between the log2-transformed expression levels of the key genes (CD44 and APP) and the estimated fractions of immune cell populations. A P-value of less than 0.05 was considered statistically significant.

### 2.8 TME characterization using ESTIMATE algorithm

To further characterize the global TME profile associated with CD44 and APP expression, the ESTIMATE algorithm was applied to calculate three key scores: stromal score, immune score, and tumor purity based on transcriptomic data. These scores reflect the abundance of stromal components, immune cell infiltration, and proportion of tumor cells, respectively. Differences in these scores between CD44/APP high- and low-expression groups were evaluated using the Mann–Whitney U test, with P < 0.05 considered statistically significant.

### 2.9 Survival analysis

To assess the prognostic value of CD44 and APP in HNSCC, survival analysis was performed using the TCGA-HNSCC cohort. Survival analysis was performed using the Kaplan-Meier method combined with the log-rank test to compare overall survival (OS) and progression-free survival (PFS) between patients with high and low expression of CD44 and APP. Hazard ratios (HR) and 95% confidence intervals (CI) were calculated using univariate Cox proportional hazards regression. A P-value < 0.05 was considered statistically significant.

### 2.10 Cells and culture conditions

The three HNSCC cell lines (CAL-27, TU177, and FaDu) along with a normal oral keratinocyte cell line (HOK) were obtained from YaJi Biological (Shanghai, China). Cells were cultured in Dulbecco’s Modified Eagle Medium supplemented with 10% fetal bovine serum, 100 U/mL penicillin, and 100 μg/mL streptomycin, and incubated at 37°C in a humidified atmosphere containing 5% CO_2_. Routine passaging was performed to ensure cells were harvested during the exponential growth phase for all experiments.

### 2.11 Quantitative real-time PCR

The expression of CD44 and APP mRNA was quantified by Quantitative real-time PCR (qRT-PCR). Total RNA was isolated from cells with TRIzol reagent (Beyotime, Cat# R0016) and reverse-transcribed into complementary DNA using 5× HiScript III qRT SuperMix (Vazyme, Cat# R323-01) according to the manufacturer’s protocol. qRT-PCR amplification was then carried out on a QuantStudio Real-Time PCR System (Thermo Fisher Scientific, USA) employing 2× Taq Pro Universal SYBR qPCR Master Mix (Vazyme, Cat# Q712-02). Gene expression was normalized to GAPDH as an internal control. The primer sequences were: for CD44, forward 5′-ACCGACAGCACAGACAGAATC-3′ and reverse 5′-GTTTGCTCCACCTTCTTGACTC-3′; for APP, forward 5′-CAAGCAGTGCAAGACCCATC-3′ and reverse 5′-AGAAGGGCATCACTTACAAACTC-3′; for GAPDH, forward 5′-GGAGCGAGATCCCTCCAAAAT-3′ and reverse 5′-GGCTGTTGTCATACTTCTCATGG-3′. Relative expression levels were determined using the 2−ΔΔCt method. Each experiment was independently repeated three times with triplicate technical replicates per run.

### 2.12 Western blot

Western blot (WB) analysis was employed to assess the protein expression levels of CD44 and APP. Total protein was extracted from the indicated cell lines. Equal amounts of protein from each sample were resolved by SDS-PAGE and subsequently transferred to a PVDF membrane. The membrane was blocked with 5% non-fat milk for 1 hour at room temperature and then probed overnight at 4 °C with specific primary antibodies: anti-CD44 (Abcam, Cat# ab157107), anti-APP (Abcam, Cat# ab32136), and anti-GAPDH (HUABIO, Cat# ET1601-4) as a loading control. After extensive washing, the membrane was incubated with appropriate horseradish peroxidase-conjugated secondary antibodies at room temperature for 1 hour. Immunoreactive bands were visualized using an enhanced chemiluminescence substrate and captured with a gel documentation system. All experiments included triplicate biological replicates.

### 2.13 Statistical methods

Continuous variables are expressed as mean ± SD. Statistical analyses were conducted with SPSS (version 25.0, IBM Corp.). Normality (Shapiro-Wilk test) and homogeneity of variance (Levene’s test) were evaluated before inter-group comparisons. For data satisfying both normality (P > 0.05) and equal variance (P > 0.05), an unpaired, two-tailed Student’s t-test was applied. Otherwise, the nonparametric Mann-Whitney U test was used. A P-value of less than 0.05 was considered statistically significant. Data visualization was performed with GraphPad Prism (version 8.0.2), and significance levels are indicated in the figures as ∗P < 0.05, ∗∗P < 0.01, and ∗∗∗P < 0.001.

## 3 Results

### 3.1 Data integration and DEG screening

**Figure 1:**
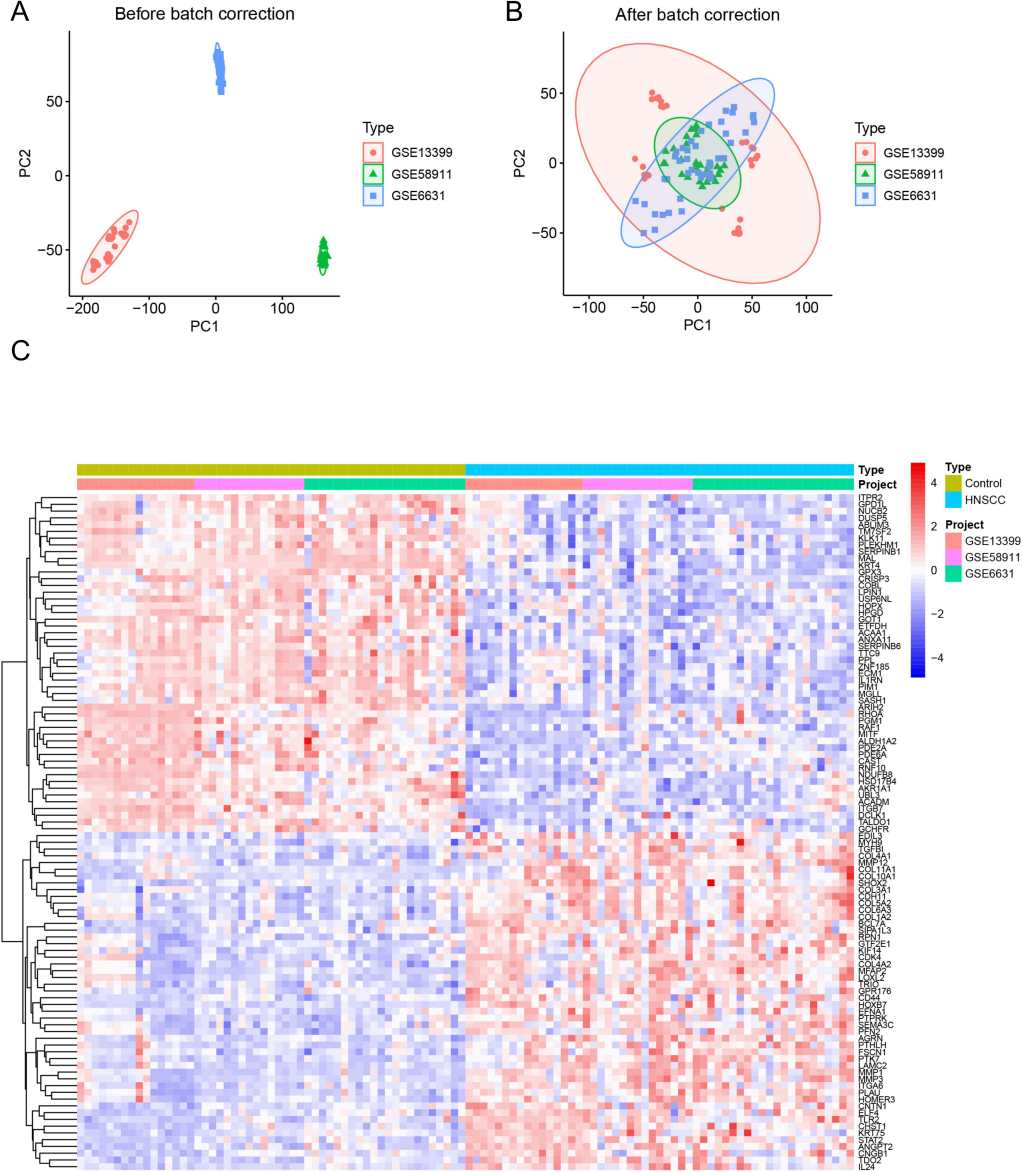
Integrated transcriptomic profiling of HNSCC reveals differential expression. **(A)** Uncorrected principal component analysis (PCA) of three transcriptomic datasets (GSE6631, GSE13399, GSE58911) reveals pronounced batch effects. **(B)** Uniform sample distribution following PCA-based batch effect correction, indicating effective harmonization. **(C)** Heatmap of the top 50 DEGs (|log_2_FC|>0.585, P < 0.05) hierarchically clustering 53 HNSCC versus 53 control samples.

For the discovery phase, three HNSCC transcriptomic datasets were retrieved from the GEO database, collectively forming a cohort of 53 HNSCC tumor tissues and 53 normal controls (**Table 1**). Expression profiles were merged and subsequently normalized to mitigate inter-dataset batch effects using PCA in R. Pre-correction analysis revealed substantial batch-driven clustering (**Figure 1A**), which was effectively alleviated post-PCA, yielding a homogenized sample distribution (**Figure 1B**). Differential expression analysis was then conducted on the batch-corrected data employing an empirical Bayes moderated linear model. Applying significance cut-offs of P < 0.05 and an |log_2_FC| greater than 0.585, we identified 1, 128 significantly DEGs (**Supplementary Table 1**). A heatmap displaying the expression patterns of the top 50 most significant DEGs illustrates the distinct transcriptional landscape of HNSCC compared to normal tissue (**Figure 1C**).

### 3.2 Identification of lactylation-associated DEGs

**Figure 2:**
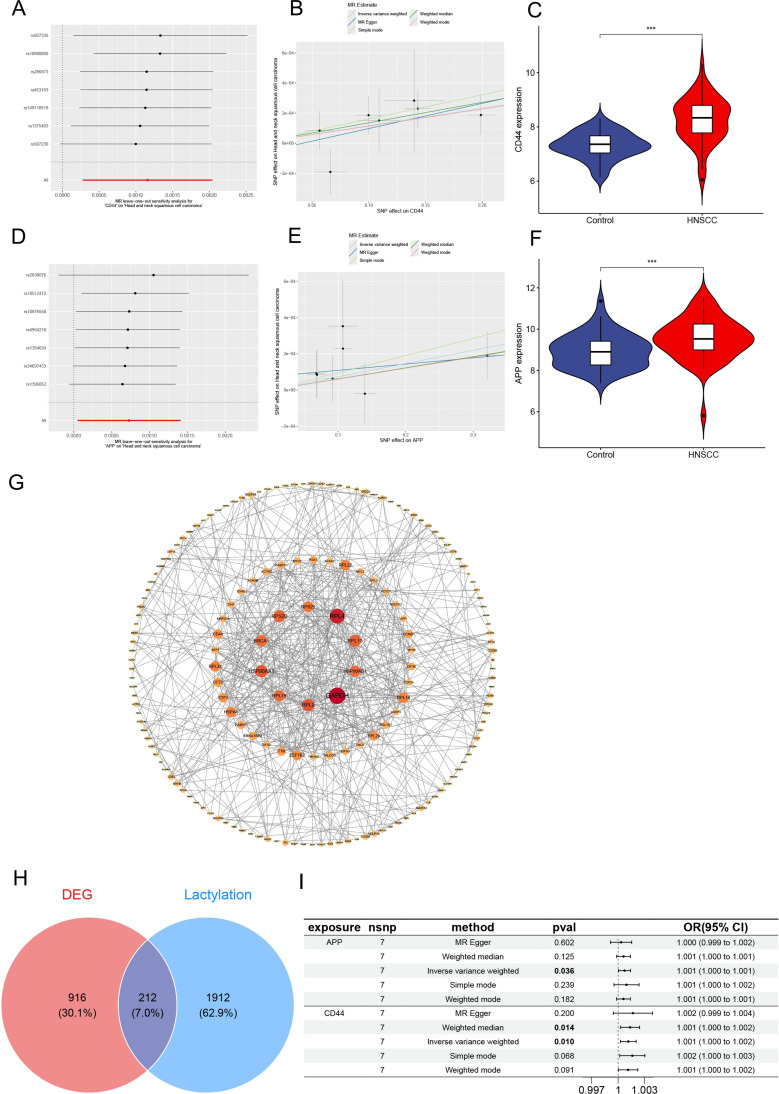
Identification of lactylation-associated causal genes in HNSCC. **(A)** Leave-one-out sensitivity analysis for CD44 in head and neck cancer GWAS. **(B)** Genetic association scatter plot for CD44. **(C)** Violin plot of CD44 expression in HNSCC vs. controls. **(D)** Leave-one-out sensitivity analysis for APP. **(E)** Genetic association scatter plot for APP. **(F)** Violin plot of APP expression in HNSCC vs. controls. **(G)** PPI network of lactylation-associated DEGs. **(H)** Venn diagram intersecting 1, 128 HNSCC-associated DEGs and 2, 124 LRGs, identifying 212 lactylation-associated DEGs. **(I)** Forest plot of MR results for prioritized genes (CD44 and APP) (*P < 0.05, **P < 0.01, ***P < 0.001.).

**Figure 3:**
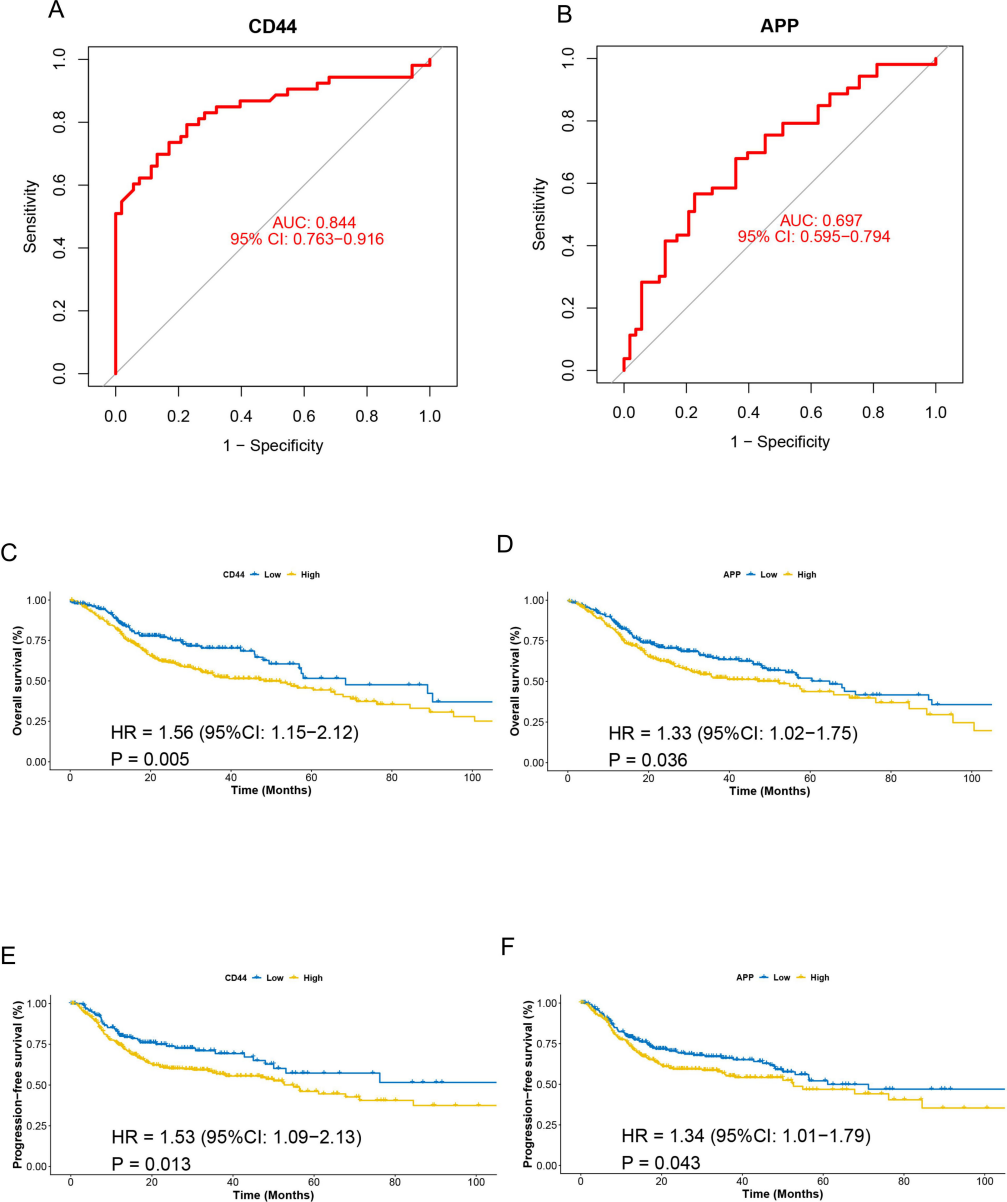
Diagnostic and prognostic value of CD44 and APP in HNSCC. **(A)** ROC curve validating CD44 diagnostic performance (AUC = 0.844). **(B)** ROC curve for APP diagnosis (AUC = 0.697). **(C)** Kaplan-Meier OS curve comparing HNSCC patients with high and low CD44 expression. **(D)** Kaplan-Meier OS curve comparing HNSCC patients with high and low APP expression. **(E)** Kaplan-Meier PFS curve comparing HNSCC patients with high and low CD44 expression. **(F)** Kaplan-Meier PFS curve comparing HNSCC patients with high and low APP expression.

From a systematic review of published literature and public databases, 2124 genes associated with lactylation modification were curated (**Supplementary Table 2**). The overlap between this set of LRGs and our identified DEGs yielded 212 intersecting genes (**Supplementary Table 3**), which we defined as lactylation-associated DEGs (**Figure 2H**). Through MR analysis under three predefined screening criteria (**Supplementary Table 5**), we identified CD44 and APP as causal risk genes that are significantly elevated in HNSCC patients. The causal relationship was supported by core MR results, including leave-one-out sensitivity plots (**Figures 2A, D**), scatter plots of genetic associations (**Figures 2B, E**), and a forest plot summarizing the IVW estimates (**Figure 2I**). Diagnostic utility assessment revealed that the expression levels of both genes could effectively discriminate HNSCC patients from healthy individuals: CD44 achieved an area under the receiver operating characteristic (ROC) curve (AUC) of 0.844 (95% CI: 0.763–0.916) (**Figure 3A**), while APP showed an AUC of 0.697 (95% CI: 0.595–0.794) (**Figure 3B**).

### 3.3 PPI network analysis identifies CD44 and APP as hub nodes

The PPI network of 212 lactylation-associated DEGs comprised a large number of interconnected genes involved in metabolic and immune regulation (**Figure 2G**). CD44 and APP were located at central positions within the network and interacted with multiple functionally important partners, including genes related to extracellular matrix organization, glycolysis, and immune cell infiltration. These observations support the role of CD44 and APP as core regulatory hubs in the metabolic–immune crosstalk of HNSCC.

### 3.4 Functional enrichment of lactylation-DEGs

**Figure 4:**
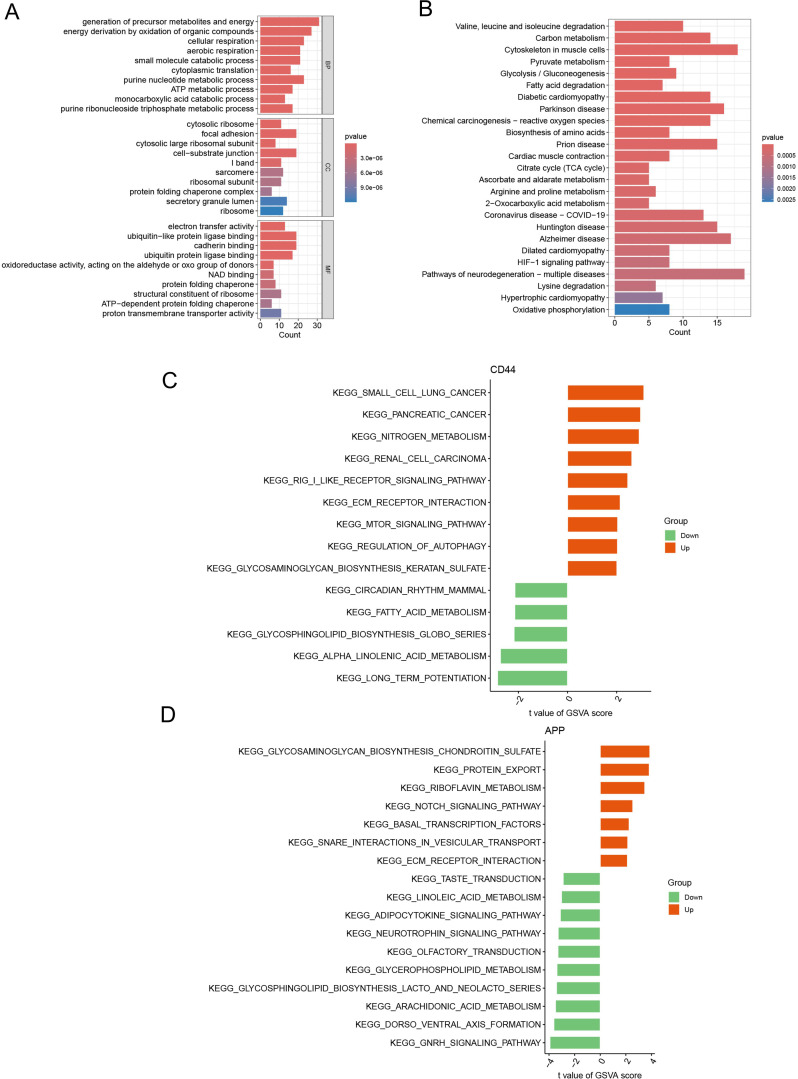
Functional enrichment of lactylation-DEGs. **(A)** Gene Ontology (GO) enrichment of 212 lactylation-DEGs. **(B)** KEGG pathway enrichment of 212 lactylation-DEGs. **(C)** GSVA of CD44 high-vs. low-expression groups. **(D)** GSVA of APP high-vs. low-expression groups.

To elucidate the functional implications of the 212 lactylation-linked DEGs, we conducted GO and KEGG pathway enrichment analyses (**Figures 4A, B**). The GO analysis identified significant enrichment in biological processes related to precursor metabolite and energy production, energy derivation from oxidation of organic compounds, cellular respiration, and purine nucleotide metabolism (**Supplementary Table 6**). KEGG pathway analysis showed that these genes were predominantly associated with pathways involving valine, leucine and isoleucine degradation, carbohydrate and energy metabolism, and HIF-1 signaling (**Supplementary Table 7**). Pathway activity profiling via GSVA revealed that samples with high CD44 expression displayed pronounced activation of pathways including Small Cell Lung Cancer, Pancreatic Cancer, Nitrogen Metabolism, Renal Cell Carcinoma, RIG-I-Like Receptor Signaling Pathway, ECM-Receptor Interaction, mTOR Signaling Pathway, Regulation of Autophagy, and Glycosaminoglycan Biosynthesis Keratan Sulfate. Conversely, pathways such as Circadian Rhythm – Mammal, Fatty Acid Metabolism, Glycosphingolipid Biosynthesis – Globo Series, Alpha Linolenic Acid Metabolism, and Long-Term Potentiation were significantly suppressed in this group (**Figure 4C**). For the APP high-expression group, significantly activated pathways comprised Glycosaminoglycan Biosynthesis Chondroitin Sulfate, Protein Export, Riboflavin Metabolism, Notch Signaling Pathway, Basal Transcription Factors, SNARE Interactions in Vesicular Transport, and ECM-Receptor Interaction. A set of pathways was notably downregulated, including Tyrosine Metabolism, Glycerolipid Metabolism, PPAR Signaling Pathway, Taste Transduction, Linoleic Acid Metabolism, Adipocytokine Signaling Pathway, Neurotrophin Signaling Pathway, Olfactory Transduction, Glycerophospholipid Metabolism, Glycosphingolipid Biosynthesis – Lacto and Neolacto Series, Arachidonic Acid Metabolism, Dorso-Ventral Axis Formation, and GnRH Signaling Pathway (**Figure 4D**). It is noteworthy that the ECM-Receptor Interaction pathway was commonly upregulated in both the CD44 and APP high-expression cohorts.

### 3.5 Assessment of immune cell infiltration in HNSCC

**Figure 5:**
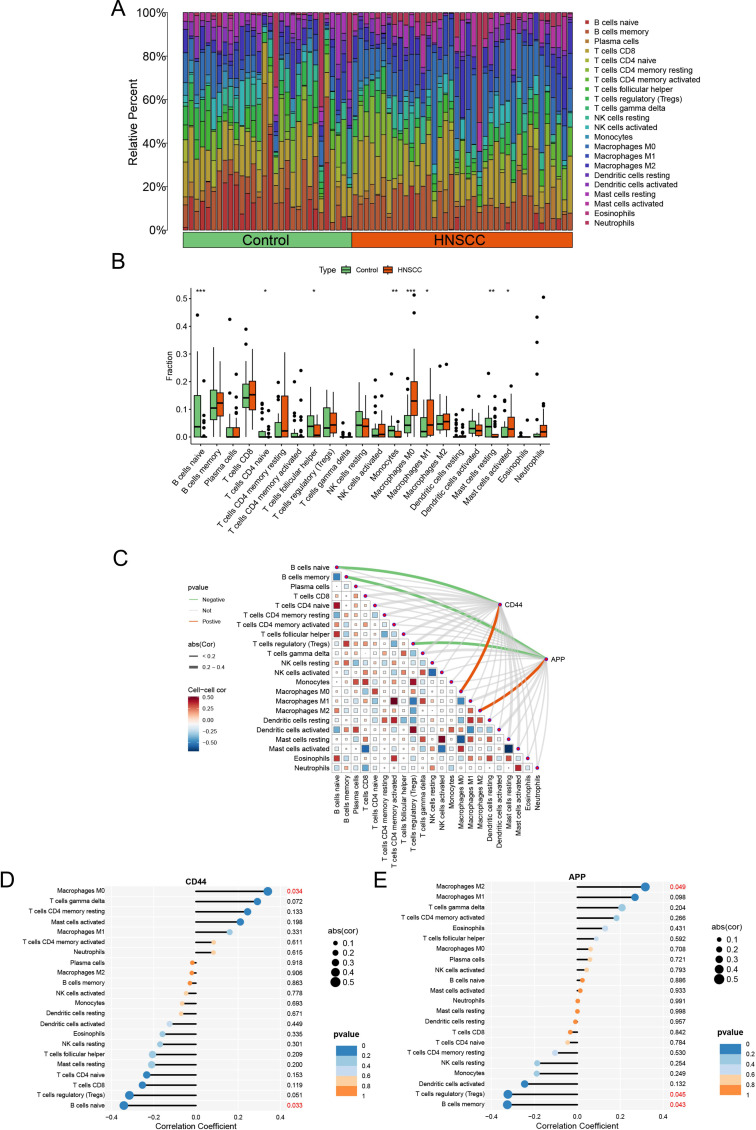
Analysis of immune cell infiltration in HNSCC. **(A)** Stacked histogram illustrating immune cell proportions across HNSCC and control groups. **(B)** Box plot demonstrating differential infiltration levels of 22 immune cell types between HNSCC and control groups. **(C)** Spearman correlation heatmap depicting associations between CD44/APP expression and immune cell subsets. **(D)** Lollipop plot visualizing correlations between 22 immune cell types and CD44 expression. **(E)** Lollipop plot visualizing correlations between 22 immune cell types and APP expression. (* P < 0.05, ** P < 0.01, *** P < 0.001).

**Figure 6:**
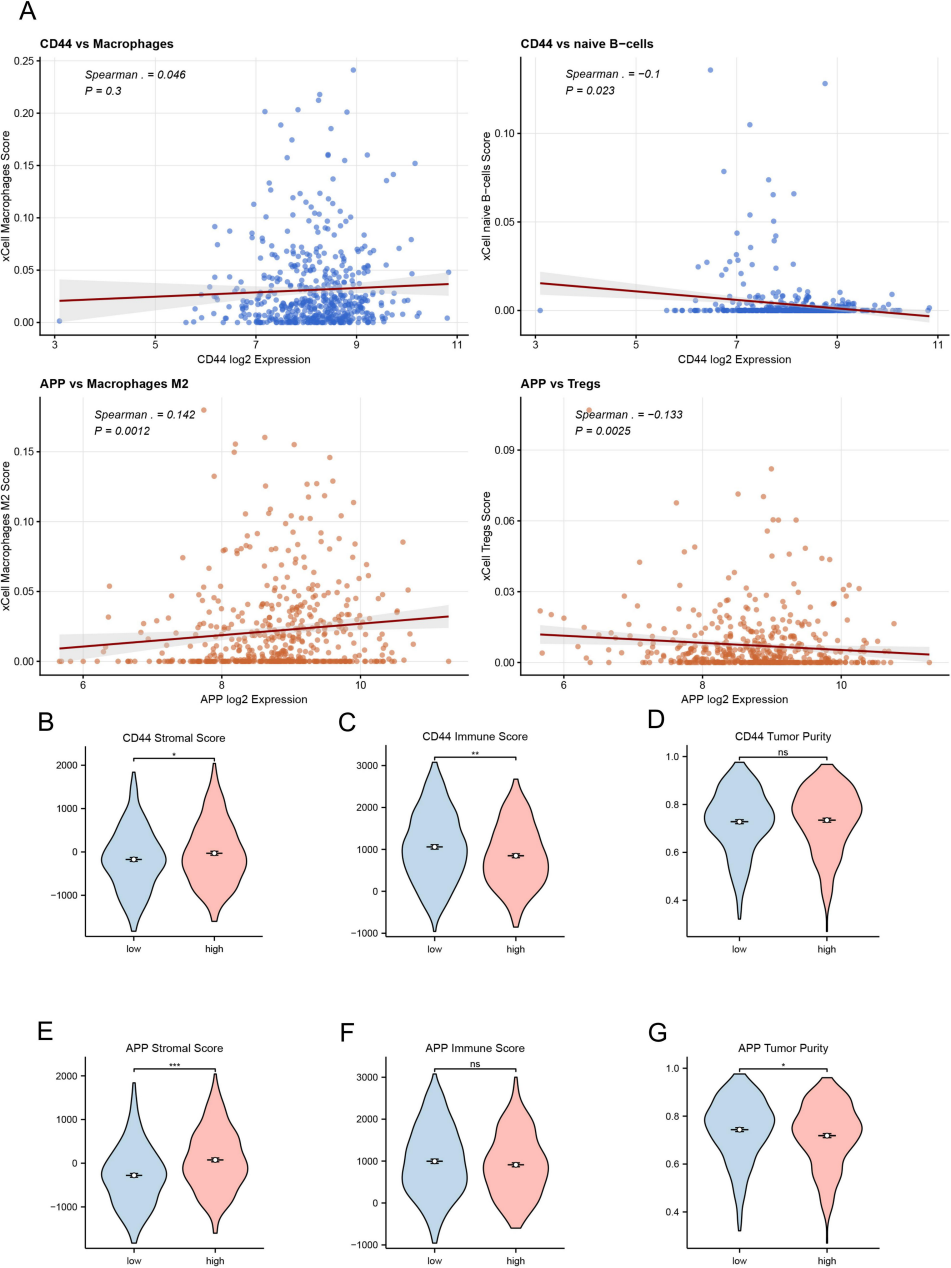
Cross-validation of immune infiltration and TME characterization. **(A)** Spearman correlation analysis between CD44/APP expression and key immune cell subsets validated by the xCell algorithm. **(B)** Stromal score between CD44 high and low expression groups. **(C)** Immune score between CD44 high and low expression groups. **(D)** Tumor purity between CD44 high and low expression groups. **(E)** Stromal score between APP high and low expression groups. **(F)** Immune score between APP high and low expression groups. **(G)** Tumor purity between APP high and low expression groups. (* P < 0.05, ** P < 0.01, *** P < 0.001, ns not significant).

Given the strong functional links of the identified HNSCC target genes to both metabolism and immunity, we next characterized the immune landscape using the CIBERSORT algorithm and examined associations between gene expression and immune infiltration. The relative abundances of 22 immune cell subsets across the cohort are visualized in **Figure 5A**. Comparative analysis demonstrated that HNSCC tissues harbored significantly higher fractions of Macrophages M0, Macrophages M1, and activated Mast cells, alongside lower proportions of naive B cells, naive CD4 T cells, follicular helper T cells, Monocytes, and resting Mast cells relative to controls (**Figure 5B**). Spearman correlation analysis further delineated specific immune associations for the key genes: CD44 expression was positively associated with Macrophages M0 infiltration but inversely correlated with naive B cells. Conversely, APP expression correlated positively with Macrophages M2 abundance, while showing negative correlations with regulatory T cells (Tregs) and memory B cells (**Figures 5C–E**). Cross-validation using the xCell algorithm further confirmed these immune correlations. Specifically, CD44 expression was positively correlated with macrophage infiltration (Spearman r = 0.046, P = 0.300) and negatively correlated with naive B-cell infiltration (Spearman r = −0.100, P = 0.023) (**Figure 6A**). APP expression was positively associated with M2 macrophage infiltration (Spearman r = 0.142, P = 0.0012) and negatively correlated with Treg abundance (Spearman r = −0.133, P = 0.0025) (**Figure 6A**). These independent computational results corroborated our CIBERSORT findings and strengthened the reliability of immune cell associations.

### 3.6 TME characteristics associated with CD44 and APP expression

To further clarify the relationship between hub gene expression and global TME composition, we performed ESTIMATE algorithm analysis. Compared with the low-expression group, patients with high CD44 expression exhibited significantly higher stromal scores and significantly lower immune scores, with no significant difference in tumor purity (**Figures 6B–D**). Similarly, high APP expression was associated with significantly elevated stromal scores and significantly reduced tumor purity, while no significant difference in immune scores was observed between groups (**Figures 6E–G**). These results indicate that upregulation of CD44 and APP is closely associated with enhanced stromal infiltration within the HNSCC microenvironment, with distinct effects on immune scores and tumor purity.

### 3.7 Prognostic value of CD44 and APP

To further evaluate the clinical prognostic significance of the identified causal genes CD44 and APP, we performed survival analysis using the TCGA-HNSCC cohort containing complete follow-up information. Kaplan-Meier survival curves demonstrated that high expression of CD44 was significantly associated with poorer overall survival in HNSCC patients (HR = 1.56, 95% CI: 1.15–2.12, P = 0.005) (**Figure 3C**). Similarly, patients with high APP expression exhibited significantly worse OS compared to those with low APP expression (HR = 1.33, 95% CI: 1.02–1.75, P = 0.036) (**Figure 3D**).

In addition to OS, we also analyzed the association between gene expression and PFS. Consistent with the OS results, high CD44 expression was significantly correlated with shorter PFS (HR = 1.53, 95% CI: 1.09–2.13, P = 0.013) (**Figure 3E**), and high APP expression was also associated with poorer PFS (HR = 1.34, 95% CI: 1.01–1.79, P = 0.043) (**Figure 3F**). These results indicate that both CD44 and APP can serve as independent prognostic biomarkers for HNSCC, further supporting their important roles in driving disease progression and recurrence.

### 3.8 Differential analysis in validation cohort

**Figure 7:**
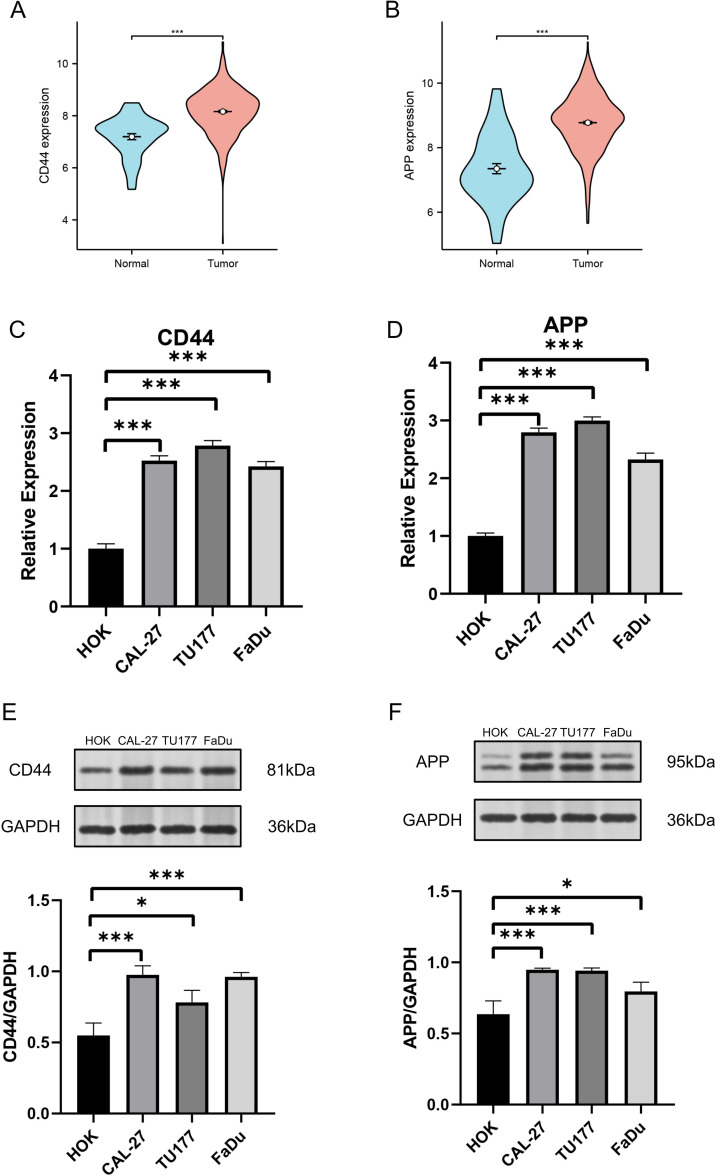
Expression validation of CD44 and APP. **(A)** Violin plot showing differential expression of CD44 between HNSCC and control tissues from the TCGA dataset. **(B)** Violin plot showing differential expression of APP between HNSCC and control tissues from the TCGA dataset. **(C)** Differential mRNA levels of CD44 in HNSCC cell lines versus the normal control cell line. **(D)** Differential mRNA levels of APP in HNSCC cell lines versus the normal control cell line. **(E)** Differential protein levels of CD44 in HNSCC cell lines versus the normal control cell line. **(F)** Differential protein levels of APP in HNSCC cell lines versus the normal control cell line. (* P < 0.05, ** P < 0.01, *** P < 0.001).

Analysis of the integrated and batch-corrected dataset from three GEO cohorts (53 HNSCC-normal pairs) revealed that CD44 and APP expression was significantly elevated in HNSCC samples relative to normal controls (**Figures 2C, F**). MR analysis further substantiated a causal genetic link, implicating high expression of these genes as risk factors for HNSCC. To independently corroborate these results, we conducted an external validation using the TCGA dataset (515 tumors vs. 44 normal tissues). Consistently, both CD44 and APP were significantly upregulated in HNSCC tissues (P < 0.05) (**Figures 7A, B**), reinforcing the evidence for their aberrant expression and likely contribution to HNSCC pathogenesis.

### 3.9 Experimental validation of hub gene expression

The expression levels of the key genes CD44 and APP were experimentally validated using qRT-PCR and WB analysis. In comparison to the normal oral keratinocyte cell line HOK, all three HNSCC cell lines (CAL-27, FaDu, and Tu177) exhibited significantly higher expression of CD44 and APP at both the transcriptional (mRNA) and translational (protein) levels (**Figures 7C–F**). These experimental results corroborate the expression patterns observed in the bioinformatic analyses. Uncropped full membrane images for the WB experiments are provided in **Supplementary Figure 1**.

## 4 Discussion

In this study, we developed and implemented a novel integrated analytical framework combining lactylation proteomics, transcriptomics, and MR to identify causal regulators of the metabolic-immune dialogue in HNSCC. Through systematic identification of 212 lactylation-related DEGs and subsequent MR analysis using extensive genetic instruments, we provide the first genetic causal evidence that CD44 and APP are risk genes for HNSCC, supported by their significant diagnostic performance. CD44 demonstrated excellent discriminative ability (AUC = 0.844), while APP showed moderate diagnostic performance (AUC = 0.697). Although the AUC for APP as a standalone biomarker indicates only moderate discriminatory power, which warrants cautious interpretation when considering it as an independent diagnostic tool, its value may be substantially enhanced when combined with CD44 or other molecular markers in a multi-marker diagnostic panel. Beyond solidifying the importance of CD44 and APP in HNSCC pathogenesis, the multi-omics causal inference strategy employed herein effectively addresses key methodological gaps: it transcends the correlative nature of traditional transcriptome studies and supplements PTMs-focused research with population-level genetic validation. The integration of genomic, transcriptomic, and PTMs data furnishes a powerful methodological blueprint for pinpointing therapeutic targets that are causally implicated and functionally versatile, exemplified by their capacity to co-regulate metabolic and immune pathways.

Cluster of Differentiation 44 (CD44) is well established as a hyaluronan receptor and a cancer stem cell marker linked to unfavorable outcomes in HNSCC (36, 37). The present MR analysis furnishes genetic evidence supporting a causal role, thereby advancing the association to a causal inference. Furthermore, our functional profiling offers a systems-level perspective that enriches the mechanistic understanding of how CD44 promotes tumorigenesis.

At the metabolic and microenvironmental level, GSVA indicated a strong link between high CD44 expression and pronounced activation of the mTOR signaling pathway, ECM-receptor interactions, and focal adhesion pathways (**Figure 4C**). These findings collectively delineate a multi-layered pro-tumorigenic network: CD44 promotes invasion directly by strengthening tumor-ECM adhesion (36), while mTOR activation acts as a master regulator of glycolysis (the Warburg effect) and protein synthesis. This concerted action likely results in abundant lactate accumulation within the TME, where lactate serves as the fundamental substrate for protein lactylation (38–40). Thus, CD44 appears to actively orchestrate, through the mTOR axis, a lactate-rich metabolic niche that facilitates its own pro-tumor functions and modulates immune activity.

On the immunoregulatory front, our correlation analysis of immune cell infiltration yielded a pivotal insight: CD44 expression was significantly and positively associated with the abundance of M0 macrophages (**Figure 5D**). This implicates CD44 primarily in the recruitment and initial mobilization of tumor-associated macrophages. In light of its concurrent activation of mTOR signaling, we hypothesize that CD44-high tumor cells may facilitate monocyte/macrophage recruitment to the tumor niche via the secretion of specific chemokines (such as CCL2) or metabolites (like lactate) (38, 41), subsequently shaping their differentiation and thereby exerting a critical influence during the nascent phase of immunosuppressive microenvironment formation. Supporting its therapeutic relevance, prior research has demonstrated that CD44 targeting enhances chemosensitivity in HNSCC cells (36), furnishing a preclinical rationale for its candidacy as a therapeutic target.

The specific mechanistic contributions of amyloid precursor protein (APP) to HNSCC pathogenesis remain incompletely understood. While earlier bioinformatic investigations have identified APP as a putative key gene in HNSCC (42), our study is the first to furnish population-level causal genetic evidence through MR. To functionally contextualize APP, we leveraged multi-tiered evidence. KEGG pathway enrichment analysis performed on the entire set of 212 lactylation-linked DEGs (which includes APP) showed significant collective enrichment for valine, leucine and isoleucine degradation and the HIF-1 signaling pathway (**Figure 4B**), implying that APP resides within a lactylation-associated network potentially co-opted for branched-chain amino acid catabolism and hypoxia adaptation. More directly, single-gene GSVA of APP (**Figure 4D**) linked its high expression to activated pathways involving protein secretion, ECM-receptor interaction, and riboflavin metabolism, suggesting that APP-high tumor cells may be adept at remodeling their microenvironment. It is noteworthy that in other cancers like glioblastoma, APP accumulation correlates with a broadly immunosuppressive milieu, lending support to a possible immunomodulatory function in tumors (43). On the immune front, correlation analysis definitively showed that APP expression positively associated with M2 macrophage infiltration (**Figure 5E**). This observation gains further relevance from recent findings that APLP2, an APP family member, is specifically upregulated in M2 macrophages and participates in regulating their polarization dynamics (44), offering a plausible mechanistic link for APP’s potential influence on the HNSCC immune landscape.

The interplay between CD44 and APP reveals a spatiotemporal and functional synergy: CD44 appears to function predominantly at the invasive tumor edge, propelling metabolic reprogramming and matrix remodeling through mTOR-ECM pathways while recruiting and priming myeloid cells like M0 macrophages. Conversely, APP likely operates within the tumor core or under adaptive stress, promoting cell survival via specialized metabolic and secretory activities and associating with an immunosuppressive, pro-fibrotic niche rich in M2 macrophages. Their complementary roles converge on pivotal stress and metabolic regulators, notably HIF-1α. This cooperative model resonates with the established complexity of cellular crosstalk in the HNSCC microenvironment (45), thereby presenting a novel, integrated framework for deciphering HNSCC progression.

Consistent with our immune infiltration findings, the ESTIMATE algorithm further revealed that high expression of CD44 and APP correlates with a more stroma-rich TME. Specifically, CD44 overexpression was associated with significantly higher stromal scores but lower immune scores, while APP upregulation was linked to elevated stromal scores and reduced tumor purity. These data indicate that both genes may promote a more desmoplastic, fibrotic microenvironment in HNSCC, which is known to restrict immune cell infiltration and contribute to immunosuppression. Notably, CD44 and APP showed distinct patterns in immune scores and tumor purity, suggesting they may exert differential effects on TME remodeling. Collectively, these results reinforce the roles of CD44 and APP as key regulators of the TME, supporting their potential as biomarkers and therapeutic targets in HNSCC.

Our findings position CD44 and APP as critical causal hubs connecting metabolic aberration and immune dysregulation in HNSCC, presenting distinct translational implications. Diagnostic and Prognostic Biomarker Potential: A composite biomarker based on CD44 and APP expression could augment existing markers like PD-L1. CD44 is a well-established independent prognostic factor in HNSCC, linked to adverse outcomes (46). Similarly, elevated APP expression correlates with advanced disease stage and poor prognosis in HNSCC (47). Notably, our survival analysis further confirmed the prognostic significance of CD44 and APP in HNSCC. High expression of both genes was significantly associated with poorer OS and PFS, which is consistent with previous clinical observations. These results provide strong evidence that CD44 and APP can be used as prognostic biomarkers to stratify HNSCC patients into different risk groups, which is crucial for guiding personalized treatment strategies. Prospective validation of this dual-gene signature for predicting patient survival, recurrence risk, and response to immune checkpoint inhibitors (ICIs) would facilitate the development of refined prognostic models. Therapeutic Targeting via Staged Intervention: A “phased intervention” strategy targeting the CD44-APP axis is conceivable. In the phase of active tumor invasion, CD44-directed therapy, supported by preclinical evidence, is relevant. During established immunosuppression, targeting APP or its related pathways may offer a new strategy. APP promotes HNSCC cell proliferation and invasion (42), and our data link its expression to M2 macrophage infiltration—a cell population that is itself a promising immunotherapeutic target in HNSCC (48). Upstream Metabolic Targeting: An alternative broad-spectrum approach involves targeting lactate production (e.g., via LDH inhibition) or the lactylation process directly. This strategy is rationalized by studies showing that suppressing LDHA-driven lactate accumulation alleviates tumor acidosis and mitigates lactate-mediated immunosuppression (49, 50). Crucially, as the direct precursor for lactylation, modulating lactate availability could influence epigenetic landscapes, such as histone lactylation, potentially yielding synergistic effects with immunotherapy (15).

Several limitations of this study should be acknowledged. First, regarding methodological constraints, potential technical confounders may arise from the complexity of integrating multi-source data and analytical pipelines. The bulk-tissue transcriptomic data used here do not capture the spatial context of different cellular subsets (e.g., M0 versus M2 macrophages); spatial transcriptomics or similar techniques are warranted to delineate cell type-specific expression patterns within the native tissue architecture. Second, at the mechanistic level, direct molecular interaction evidence supporting the core “metabolic-immune crosstalk” hypothesis is still lacking. Although MR analysis provides genetic evidence for causality, definitive proof that CD44 and APP are direct substrates of protein lactylation and a detailed understanding of their lactylation-dependent functions await experimental confirmation via co-immunoprecipitation coupled with mass spectrometry and site-specific mutagenesis studies. Furthermore, the pro-tumorigenic roles of CD44 and APP and their therapeutic druggability require rigorous *in vivo* validation using genetic approaches and patient-derived xenograft or humanized mouse models. Third, several aspects of clinical translation and generalizability remain to be addressed. Our expression analyses were not stratified by HNSCC subsites (oral, laryngeal, oropharyngeal), which exhibit distinct etiological backgrounds, HPV prevalence, and clinical prognosis. The current MR analysis utilized predominantly European ancestry GWAS data, and its generalizability needs to be tested in multi-ethnic cohorts. Regarding immuno-oncology applications, although we have characterized TME composition using CIBERSORT, xCell, and ESTIMATE algorithms, additional analyses—including TIDE-based prediction of immunotherapy responsiveness, Immunophenotype Score quantification, and systematic co-expression profiling of CD44/APP with key immune checkpoint molecules (e.g., PD-1, PD-L1, CTLA-4, LAG-3, TIM-3)—would provide more direct evidence for the immunotherapeutic implications of CD44 and APP dysregulation. Additionally, a comprehensive prognostic risk signature based on the 212 lactylation-related DEGs, rather than single-gene analysis, may offer superior risk stratification and warrants dedicated future investigation. These analyses are being prioritized in our ongoing work and are expected to further elucidate the therapeutic potential of targeting the CD44-APP axis, particularly in combination with immunotherapy.

## 5 Conclusion

In conclusion, we established an integrative analytical framework that merges lactylomics, transcriptomics, and MR, and pioneered its application to uncover causal drivers in HNSCC. Utilizing this approach, we pinpointed CD44 and APP as causal risk genes for this malignancy. Subsequent systems-level investigations delineate that CD44 and APP act as central regulators of the metabolic-immune crosstalk, converging at the interface of metabolic rewiring and immune dysfunction within the TME and are associated with HNSCC progression. Collectively, our work provides a deeper, systems-level understanding of HNSCC pathogenesis grounded in causal inference, and lays a critical theoretical groundwork for future development of biomarkers and therapies targeting the lactylation axis and the interconnected metabolic-immune network.

## Data Availability

The original contributions presented in the study are included in the article/**Supplementary Material**. Further inquiries can be directed to the corresponding author.
