# Supplementary figures and images for: Metabolic-immune crosstalk in head and neck squamous cell carcinoma: CD44 and APP identified as causal therapeutic targets via integrated lactylation-Mendelian randomization analysis
Source: Front Immunol. 2026 May 28;17:1808455. doi: 10.3389/fimmu.2026.1808455 (PMC13254516; doi:10.3389/fimmu.2026.1808455)

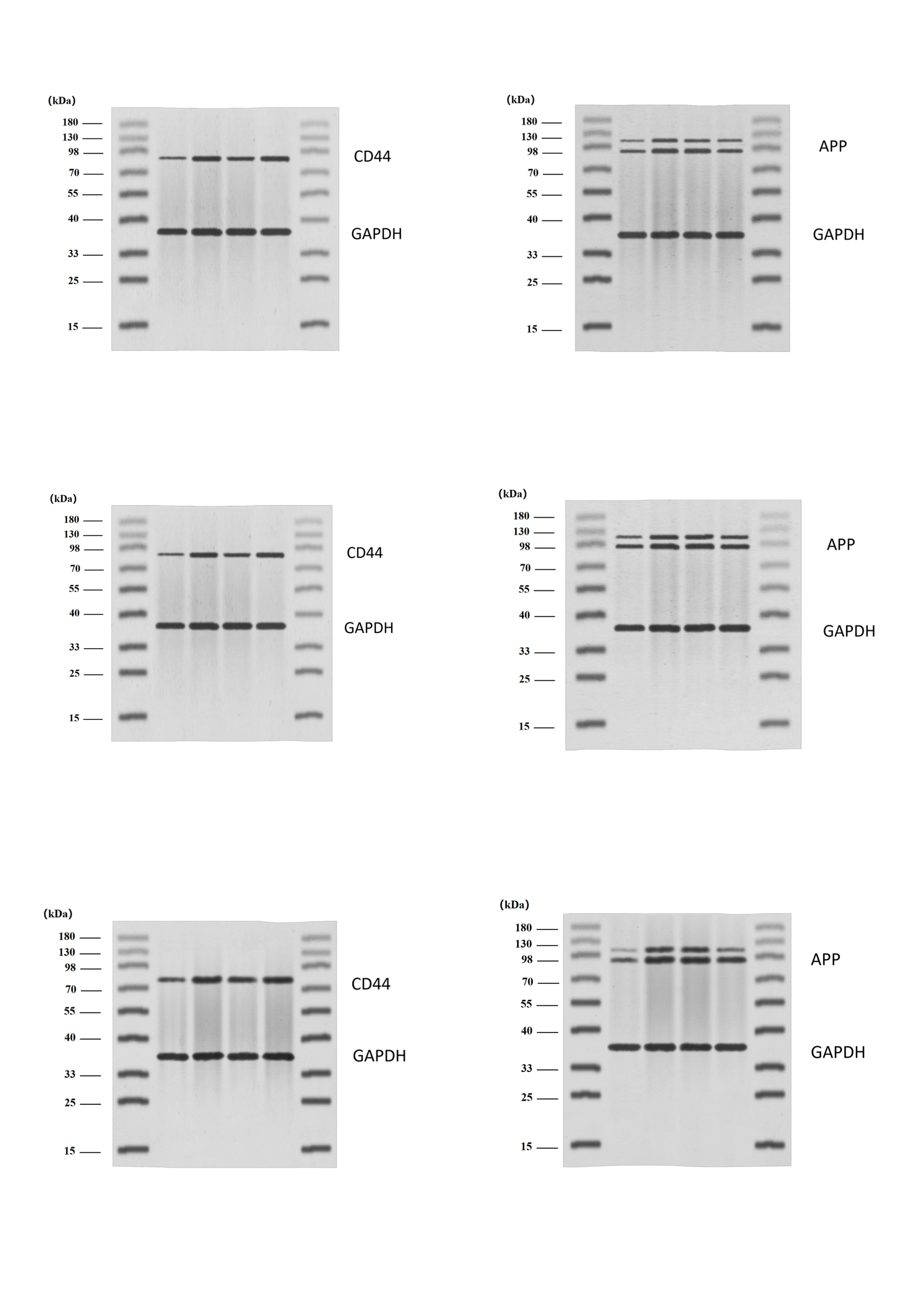

Supplement: Supplementary file 1 [file Image1.jpeg]
